# Supplementary material for: Whole genome sequencing of extended-spectrum β-lactamase genes in Enterobacteriaceae isolates from Nigeria
Source: PLoS One. 2020 Apr 14;15(4):e0231146. doi: 10.1371/journal.pone.0231146 (PMC7156064; doi:10.1371/journal.pone.0231146)
Supplement: S2 Table — (DOCX) [file pone.0231146.s002.docx]

| **Ids** | **Org** | **CAZ** | **CTX** | **AMC** | **FOX** | **FEP** | **ATM** | **MEM** | **CIP** | **AK** | **ETP** | **TZP** | **C** | **LEV** | **W** |
| --- | --- | --- | --- | --- | --- | --- | --- | --- | --- | --- | --- | --- | --- | --- | --- |
| 5823 | *E.coli* | r | r | 16r | 23s | 13r | 9r | 30s | r | 18s | 29s | 19i | 24s | 9r | R |
| 4641 | *E. asburiae* | 11r | r | 10r | R | 16r | 15r | 32s | 15r | 21s | 29s | 20s | R | 25s | R |
| 1337 | *K.pneumoniae* | r | r | 17r | 25s | 15r | 10r | 31s | 19s | 18s | 30s | 17i | 25s | 23s | R |
| 4507 | ***E. hormaechei*** | 10r | r | r | r | 17r | 13r | 32s | 19i | 21s | 31s | 18i | 24s | 26s | R |
| 1259 | *E. asburiae* | 12r | r | 10r | r | 16r | 15r | 31s | 15r | 21s | 28s | 19i | R | 17r | R |
| 5832 | *E. asburiae* | 10r | r | 9r | r | 14r | 13r | 30s | 14r | 20s | 28s | 19i | R | 18r | R |
| 4354 | *E.coli* | 12r | r | 20s | 22s | 16r | 14r | 31s | r | 17i | 30s | 23s | 22s | r | R |
| 5854 | *K.pneumoniae* | 11r | r | 15r | 24s | 18r | 13r | 29s | r | 19s | 29s | 18i | 27s | 10r | R |
| 5089 | *E.coli* | r | r | 16r | 16r | 16r | r | 35s | r | 20s | 25s | 20s | R | r | R |
| 1628 | *K.pneumoniae* | r | r | 16r | 24s | 15r | 10r | 31s | 19s | 20s | 30s | 18i | 24s | 24s | R |
| 4594 | ***E. hormaechei*** | 10r | r | r | r | 16r | 13r | 32s | r | 20s | 31s | 18i | 20s | r | R |
| 3467-2 | *E.coli* | 11r | r | 19s | 25s | 19r | 12r | 35s | r | 21s | 30s | 20s | 25s | 10r | R |
| 3600 | *k.pneumoniae* | 11r | r | 14r | 25s | 17r | 12r | 30s | 21i | 20s | 29s | 17i | R | 23s | R |
| 3682 | *k.pneumoniae* | r | r | 15r | 21s | 15r | r | 30s | r | r | 27s | 16r | 19s | r | R |
| 3264 | *k.pneumoniae* | 11r | r | 16r | 26s | 17r | 14r | 30s | r | 19s | 29s | 17i | 26s | 11r | R |
| 263 | *K.pneumoniae* | r | r | 17r | 24s | 14r | 9r | 30s | 19s | 20s | 29s | 18i | 25s | 24s | R |
| 4595 | *k.pneumoniae* | 12r | r | 19s | 25s | 18r | 14r | 31s | 19i | 22s | 29s | 22s | R | 23s | R |
| 3397 | *E.coli* | 11r | r | 18r | 24s | 17r | 12r | 32s | r | 19s | 31s | 18i | 24s | r | R |
| 11 | *E.coli* | 15r | r | **8r** | 25s | 22i | 14r | 34s | r | 22s | 35s | 22s | R | r | R |
| 1476 | *E. cloacae* | 12r | r | 9r | r | 19r | 16r | 32s | 21i | 21s | 30s | 21s | R | 26s | R |
| Y3 | *K.pneumoniae* | 9r | r | 14r | 23s | 15r | 11r | 29s | 16r | 19s | 22s | 17i | R | 22s | R |
| D30-04 | *K.pneumoniae* | 17r | r | 19s | 27s | 23i | 19r | 34s | 25s | 24s | 33s | 20s | 25s | 27s | R |
| 838 | *E.coli* | 22s | r | 22s | 16r | 19r | 21i | 32s | r | 23s | 33s | 23s | R | r | R |
| 2822 | *k.pneumoniae* | 10r | r | 16r | 23s | 19r | 12r | 31s | r | 22s | 31s | 17i | R | r | R |
| 2821 | *E.coli* | r | r | 22s | 20s | 9r | r | 33s | r | 22s | 28s | 20s | R | r | R |
| 4374 | *E.coli* | 22s | r | 19s | 24s | 24s | 18r | 32s | r | 20s | 30s | 22s | R | r | R |
| 3608 | ***A.hermannii*** | 14r | r | 20s | 28s | 19r | 17r | 31s | 21i | 21s | 31s | 22s | R | 26s | R |
| 4502 | *K.pneumoniae* | 10r | r | 18r | 21s | 18r | 10r | 32s | r | 19s | 30s | 17i | 19s | r | R |
| 2654 | *E. cloacae* | 13r | r | 9r | r | 21i | 16r | 33s | 22s | 22s | 30s | 21s | R | 25s | R |
| 2668 | *E.coli* | 10r | r | 14r | 25s | 17i | 13s | 31s | r | 20s | 32s | 14r | R | 9r | R |
| 157 | *k.pneumoniae* | r | r | 14r | 21s | 13r | 10r | 29s | 18r | 19s | 27s | 18i | 24s | 22s | R |
| Q6 | *E.cloacae* | 9r | r | r | r | 17i | 10r | 30s | 20i | 19s | 23s | 16r | R | 24s | r |
| 2781 | ***E.hormaechei*** | 12r | r | 10r | r | 19r | 13r | 33s | r | 20s | 30s | 18i | R | 9r | R |
| 872 | *K.pneumoniae* | r | r | r | 20s | 9r | r | 23i | r | 21s | 16r | r | R | r | R |
| 852 | *k.pneumoniae* | r | r | 10r | r | 11r | r | 14r | r | r | 11r | r | 14r | r | 17i |
| 1337LF | *K.pneumoniae* | r | r | r | 19s | 17r | r | 23s | r | 20s | 15r | r | R | r | R |
| 3442 | *E.coli* | r | r | 9r | r | r | r | 30s | r | 19s | 26s | r | 24s | r | R |
| 2840 | *k.pneumoniae* | 16r | r | 15r | 25s | 17r | 13r | 32s | 33s | 21s | 30s | 19i | R | 31s | R |
| 852K | *k.pneumoniae* | r | r | 11r | r | 12r | r | 16i | r | r | 12r | r | 17s | r | 18s |
| 2644 | ***E..hormaechei*** | 10r | r | 10r | r | 14r | 12r | 30s | r | 19s | 29s | 18i | R | 9r | R |
| C2 | *k.pneumoniae* | r | r | 13r | 22s | 17r | r | 31s | r | 22s | 28s | 14r | 25s | r | R |
| 570 | *E.coli* | r | r | 15r | 17r | 15r | 8r | 35s | r | 22s | 32s | 20s | 18s | r | R |
| A3 | *k.pneumoniae* | 12r | r | 17r | 24s | 20r | 15r | 30s | r | 20s | 30s | 18i | 26s | 10r | R |
| 12 | *E.coli* | r | r | 9r | r | r | r | 32s | r | 20s | 25s | r | 19s | r | R |
| C8 | *k.pneumoniae* | r | r | 14r | 22s | 18r | r | 29s | 8r | 23s | 28s | 14s | 23s | 10r | R |
| 2471 | *k.pneumoniae* | r | r | 14r | 22s | 19r | r | 33s | r | 20s | 29s | 14r | R | r | R |
| 3471 | *E.coli* | r | r | 20s | 16r | 12r | r | 29s | r | 22s | 27s | 21s | R | r | R |
| 2580 | ***C.werkmanii*** | 9r | r | 12r | r | 19r | r | 37s | r | 20s | 33s | 20s | R | r | R |
| UI2 | *k.pneumoniae* | r | r | 14r | 25s | 14r | 10r | 29s | 34s | 21s | 27s | 14r | 26s | 33s | R |
| C01 | *k.pneumoniae* | r | r | 16r | 25s | 14r | 10r | 29s | 32s | 22s | 27s | 15r | 27s | 32s | R |
| A01 | *E.coli* | r | r | 19s | 18r | 15r | r | 30s | r | 20s | 26s | 22s | R | r | R |
| 3688 | ***E.hormaechei*** | r | r | 12r | r | 14r | r | 32s | r | 22s | 20r | 18i | R | r | R |
| 3628 | ***E.hormaechei*** | r | r | r | r | 11r | r | 26s | r | 20s | 23i | 17i | R | r | R |
| R2 | *K.pneumoniae* | r | r | 18r | 23s | 14r | r | 31s | r | 21s | 27s | 18i | R | r | R |
| R3 | *E.cloacae* | r | r | 9r | r | 10r | r | 30s | r | 13r | 22s | 13r | R | r | R |
| C02 | *E.coli* | 8r | r | 19s | 19s | 13r | r | 31s | r | 21s | 30s | 24s | R | r | R |
| 1643K | *K.pneumoniae* | 13r | r | 16r | 25s | 19r | 13r | 32s | r | 21s | 32s | 19i | 28s | 10r | R |
| 3385 | *E. coli* | 9r | r | 20s | 21s | 15r | 9r | 33s | r | 23s | 30s | 22s | r | r | R |
| 2^14/05^ | *E.cloacae* | 12r | r | 9r | r | 19r | 15r | 33s | 22s | 21s | 30s | 20s | r | 27s | R |
| C4 | *P. mirabilis* | 26s | r | 21s | 27s | 21i | 34s | 34s | r | 24s | 34s | 26s | r | r | r |

CAZ-Ceftazidime 10µg, CTX-Cefotaxime 5µg, AMC- Amoxicillin-clavulanic acid 30µg, FOX-Cefoxitin 30µg, FEP- Cefepime-30µg, ATM- Azetronam 30µg, MEM- Meropenem10µg, CIP- Ciprofloxacin 5µg, AK- Amikacin 30µg, ETP- Ertapenem 10µg, TZP- Piperacillin-tazobactam 36µg, C- Chlramphenicol 30µg, LEV- Levofloxacin 5µg, W-Trimethoprim 5µg
