# Supplementary material for: Whole genome sequencing of extended-spectrum β-lactamase genes in Enterobacteriaceae isolates from Nigeria
Source: PLoS One. 2020 Apr 14;15(4):e0231146. doi: 10.1371/journal.pone.0231146 (PMC7156064; doi:10.1371/journal.pone.0231146)
Supplement: S3 Table — (DOCX) [file pone.0231146.s003.docx]

| Number | Hospital | Identification | Organism | Source | Sex | Age |
| --- | --- | --- | --- | --- | --- | --- |
| 1 | UBTH | 5823 | *E.coli* | URINE | F | 58 |
| 2 | UBTH | 4641 | *E. asburiae* | URINE | M | AD |
| 3 | UBTH | 1337 | *K.pneumoniae* | SWAB | M | AD |
| 4 | UBTH | 4507 | ***E. hormaechei*** | PLEURAL ASPIRATE | M | 38 |
| 5 | UBTH | 1259 | *E. asburiae* | URINE | F | 57 |
| 6 | UBTH | 5832 | *E. asburiae* | URINE | F | 81 |
| 7 | UBTH | 4354 | *E. coli* | URINE | F | 29 |
| 8 | UBTH | 5854 | *K. pneumoniae* | URINE | M | AD |
| 9 | UBTH | 5089 | *E.coli* | URINE | F | AD |
| 10 | UBTH | 1628 | *K.pneumoniae* | URINE | F | 15 |
| 11 | UBTH | 4594 | ***E. hormaechei*** | URINE | M | AD |
| 12 | CH | C4 | *P.mirabilis* | URINE | M | 46 |
| 13 | UBTH | 3467-2 | *E.coli* | URINE | M | 60 |
| 14 | UBTH | 3600 | *K.pneumoniae* | URINE | M | 33 |
| 15 | UBTH | 3682 | *K.pneumoniae* | URINE | M | 72 |
| 16 | UBTH | 3264 | *K.pneumoniae* | URINE | F | AD |
| 17 | UBTH | 263 | *K.pneumoniae* | BLOOD | F | 5 DAYS |
| 18 | UBTH | 4595 | *K.pneumoniae* | URINE | F | AD |
| 19 | UBTH | 3397 | *E.coli* | WOUND SWAB | F | AD |
| 20 | UBTH | 11 | *E.coli* | URINE | M | AD |
| 21 | CH | 214-05 | *E.cloacae* | WOUND SWAB | F | 80 |
| 22 | UBTH | 1476 | *E.cloacae* | URINE | M | 63 |
| 23 | UBTH | Y3 | *K.pneumoniae* | UIS | N/A | N/A |
| 24 | CH | D30-04 | *K.pneumoniae* | WOUND | M | 62 |
| 25 | UBTH | 838 | *E.coli* | URINE | M | AD |
| 26 | UBTH | 2822 | *K.pneumoniae* | WOUND SWAB | M | 7 MTHS |
| 27 | UBTH | 2821 | *E.coli* | CATHETER TIP | M | 45 |
| 28 | UBTH | 4374 | *E.coli* | ECS | N/A | N/A |
| 29 | UBTH | 4502 | *K.pneumoniae* | WOUND SWAB | M | AD |
| 30 | UBTH | 2654 | *E.cloacae* | URINE | M | AD |
| 31 | UBTH | 2668 | *E.coli* | WOUND SWAB | M | AD |
| 32 | UBTH | 157 | *K.pneumoniae* | BLOOD | M | 5 DAYS |
| 33 | UBTH | Q6 | *E.cloacae* | UIS | N/A | N/A |
| 34 | UBTH | 2781 | ***E. hormaechei*** | URINE | F | 28 |
| 35 | UBTH | 872 | *K.pneumoniae* | ECS | F | 46 |
| 36 | UBTH | 852 | *K.pneumoniae* | URINE | M | 28 |
| 37 | UBTH | 1337LF | *K.pneumoniae* | URINE | M | 56 |
| 38 | UBTH | 3442 | *E.coli* | URINE | M | AD |
| 39 | UBTH | 2840 | *K.pneumoniae* | URINE | F | AD |
| 40 | UBTH | 852K | *K.pneumoniae* | URINE | M | 28 |
| 41 | UBTH | 2644 | ***E .hormaechei*** | URINE | M | 60 |
| 42 | CH | C2 | *K.pneumoniae* | URINE | F | 28 |
| 43 | UBTH | 570 | *E.coli* | URINE | M | 70 |
| 44 | CH | A3 | *K.pneumoniae* | WOUND SWAB | F | 70 |
| 45 | UBTH | 12 | *E.coli* | URINE | M | AD |
| 46 | CH | C8 | *K.pneumoniae* | URINE | F | 27 |
| 47 | UBTH | 2471 | *K.pneumoniae* | URINE | M | 62 |
| 48 | UBTH | 3471 | *E.coli* | URINE | F | 53 |
| 49 | UBTH | 2580 | ***C. werkmanii*** | URINE | F | 76 |
| 50 | UBTH | UI2 | *K.pneumoniae* | URINE | F | 33 |
| 51 | CH | C01 | *K.pneumoniae* | HVS | F | 29 |
| 52 | CH | A01 | *E.coli* | URINE | F | 19 |
| 53 | UBTH | 3688 | ***E. hormaechei*** | URINE | M | 82 |
| 54 | UBTH | 3628 | ***E. hormaechei*** | URINE | M | AD |
| 55 | IUTH | R2 | *K.pneumoniae* | UIS | N/A | N/A |
| 56 | IUTH | R3 | *E.cloacae* | UIS | N/A | N/A |
| 57 | CH | C02 | *E.coli* | HVS | F | 29 |
| 58 | UBTH | 1643K | *K.pneumoniae* | Urine | F | 78 |
| 59 | UBTH | 3608 | ***A.hermanii*** | Wound swab | F | 77 |
| 60 | UBTH | 3385 | *E. coli* | Catheter tip | F | 58 |
