# Supplementary material for: Whole genome sequencing of extended-spectrum β-lactamase genes in Enterobacteriaceae isolates from Nigeria
Source: PLoS One. 2020 Apr 14;15(4):e0231146. doi: 10.1371/journal.pone.0231146 (PMC7156064; doi:10.1371/journal.pone.0231146)
Supplement: Attachment — Submitted filename: Response to reviewers.docx [file pone.0231146.s005.docx]

Additional Editor’s comments

This manuscript is of great interest for us, please address the questions and suggestions offered by the reviewers.

Journal requirements:

When submitting your revision, we need you to address these additional requirements.

 1. Please ensure that your manuscript meets PLOS ONE's style requirements, including those for file naming. The PLOS ONE style templates can be found at

http://www.journals.plos.org/plosone/s/file?id=wjVg/PLOSOne_formatting_sample_main_body.pdf and <http://www.journals.plos.org/plosone/s/file?id=ba62/PLOSOne_formatting_sample_title_authors_affiliations.pdf>

**The manuscript has been re-written to meet PLOS ONE’s style requirements including those for file naming.**

2. Please amend either the title on the online submission form (via Edit Submission) or the title in the manuscript so that they are identical.

**The title on the online submission form and the manuscript are now identical.**

3. We note that you have stated that you will provide repository information for your data at acceptance. Should your manuscript be accepted for publication, we will hold it until you provide the relevant accession numbers or DOIs necessary to access your data. If you wish to make changes to your Data Availability statement, please describe these changes in your cover letter and we will update your Data Availability statement to reflect the information you provide.

**All necessary data are made available already and relevant accession numbers are provided in the revised manuscript**.

4. Please include captions for your Supporting Information files at the end of your manuscript, and update any in-text citations to match accordingly. Please see our Supporting Information guidelines for more information: <http://journals.plos.org/plosone/s/supporting-information>

**The supporting information files have been worked on and in-text citations updated to match accordingly**

Review Comments to the Author

Reviewer #1: Jesumirhewe and collaborators present an interesting manuscript with the general objective of determine the frequency of ESBL genes in multidrug resistant human clinical Enterobacteriaceae isolates from Edo state Nigeria and to characterize the resistance mechanisms. Thank you for being able to contribute to it before your presentation to the scientific community. Therefore, I suggest that the following questions be modified, deleted, or explained.

Line 1. I think authors should change in the title: Whole genome sequence of extended-spectrum β-lactamase genes in Enterobacteriaceae isolates from Nigeria.

**The title of the article has been changed to Whole genome sequencing of extended-spectrum β-lactamase genes in *Enterobacteriaceae* isolates from Nigeria**

Improve the Abstract and Introduction. ESBL concern in both healthcare and community settings

**The abstract and introduction has been improved on. Concern about ESBL in both healthcare and community settings have been highlighted**

Line 16, 64. “This study aimed at determining the frequency of ESBL genes in multidrug resistant human clinical Enterobacteriaceae isolates from Edo state Nigeria” using whole genome sequencing

**The sentence has been edited in the revised manuscript**

Line 33. I suggest to describe Ambler classification of beta-lactamases and groups of CTX-M on the introduction.

**Ambler classification of beta-lactamases have been described and groups of CTX-M mentioned on the introduction**

Line 51. I would rather see table 1 summarized in a Figure instead of a table. I suppose it would be better demonstrated. The three hospitals are at a different level of care?

**Table 1 is now presented as a figure. The 3 hospitals are tertiary healthcare institutions.**

Why did you decide to include the strains of unidentified source; Teaching Hospital, Benin n=12, and Okada n=4

**I included strains of unidentified source [Data not available} because some of these strains are important i.e had the esbl resistant gene and associated resistant determinants. Removing them from the number of isolates would not give a true picture of the frequency of resistant determinants present in the sample size**

Line 74. “Flurometer” Fluorometer

**Fluorometer spelling corrected**

Line 94. The authors reported that (41%) were positive for the ESBL phenotypic test and had ESBL genes detected. Please explain the results of Pret3577 and EC11, where no resistance pattern to B lactams was observed. In strains with ESBL and carbapenemase, the ESBL screening and confirmation performs well?

**There was a mix-up in our earlier report. Isolates that were positive for the ESBL phenotypic test and had ESBL genes detected are sixty [40%]. Pret3577 was sensitive to the ESBL phenotypic test but had the ESBL gene detected by WGS.EC 11 was resistant to both ceftazidime and cefotaxime. The ESBL screening and confirmation performed well in strains with ESBL and carbapenemase**

Line 115. Table 4. Please highlight in results the presence of carbapenemases NDM and Oxa-48 genes in strains Kp_852 ST15, Kp_852K ST15, Kp_872 ST340 (First cases described?)
I recommend to improve tables, columns of ST set in the beginning
Have all the sequences been deposited at GenBank?
Is it possible probable clonal spread of the ESBL strains Intra or inter-hospital
Limitations must be included
I suggest to include virulence factors and mobile genetic elements associated with ESBL and phylogeny based on core genome MLST.
I recommend to highlight the WGS findings (figures), highlight the emerging global high risk clones, and to present this manuscript in a better fashion.

**The carbapenemases have been earlier reported along with a few other isolates that are not ESBL producers as indicated in the manuscript with tracked changes. The aim of this study was to determine the frequency of ESBL genes in multidrug resistant human clinical *Enterobacteriaceae* isolates from Edo state Nigeria. Tables have been improved on. S1 Table gives the characteristics of the ESBL isolates. Sequences have been deposited at the GenBank and accession numbers included on S1 Table.Yes there is a possibility of probable clonal spread of the ESBL strains especially intra-hospital. The WGS findings have been re-presented. A limitation in this study is to conclude on clonal bacterial transmission between the ESBL *Enterobacteriaceae* isolates that included seven groups of different organisms. However,** **the presence of different plasmid replicon types in ESBL producing isolates reveals their importance in the dissemination of these resistance genes.**

Reviewer #2: The manuscript evaluates the frequency of ESBL genes in multidrug resistant human clinical Enterobacteriaceae isolates from Edo state Nigeria and characterize the resistance mechanisms. The research is correctly conducted, and the conclusions are supported by the data. However, results are not well exposed and are difficult to understand.

**The results have been represented and are now easy to understand**

Materials and method
Line 66. The phenotypic method used, is not useful for microorganisms with Amp C , so some ESBL producers could not be included, and this could be considered as a limitation

**The phenotypic method used was based on CLSI guidelines. All ESBL producers detected phenotypically were confirmed in the WGS.Only one isolate had ESBL detected by WGS but not detected phenotypically**

Table 1. Use italics to Enterobacteriaceae. This table does not present summarized data and contains partial results. Include n values

**Table 1 has been re-presented as a figure**

Results: Include summary tables or figures

**Results have been re-presented**

Table 2. Present information by isolated microorganism or at least the predominant ones. cite the % and include the n, also include the denominator of isolates tested for each antimicrobial listed

**Information has been re-presented showing the % of ESBL isolates resistant to the various antibiotics with n included and the denominator of isolates tested for each antimicrobial listed**

Line 93. Include n value

**n value has been included**

Table 3. summarize with MIC 50 and MIC 90 values. This information must be included as supplementary material**.**

**The MIC values are included as supporting information now.The MIC values are presented only for the 60 ESBL isolates that are multidrug resistant. MIC values were not determined for the entire 218 enterobacteriaceae isolates. The E-test has been reported to be considered a reliable method to determine antimicrobial susceptibility testing and it gives results which are at least as accurate as those obtained by the broth dilution method. As our ESBL isolates are not up to 100 and the Enterobacteriaceae isolates are a collection of 7 different group of isolates calculating MIC50 and MIC90 may not be applicable here**

Line 99 -114 summarize using tables or figures

**Line 99-114 has been summarized and tables represented. The genetic characteristics of the ESBL isolates are presented in the supporting information with other details.**

Line 139. How many isolates were studied by MLST? Include n values

**All the multidrug resistant isolates that are ESBL producers were studied by MLST, n=60, The sensitive isolate that genotypically had ESBL detected was also studied by MLST, n=1. Some of the isolates did not have available sequence types.**

What was the resistance mechanism involved in meropenem resistance?
None S. marcescens isolate had ESBL genes?

**No S. marcescens isolate had ESBL genes.** **The carbapenemases have been earlier reported along with a few other isolates that are not ESBL producers as indicated in the manuscript with tracked changes. The aim of this study was to determine the frequency of ESBL genes in multidrug resistant human clinical *Enterobacteriaceae* isolates from Edo state Nigeria.**
