# Supplementary material for: Whole genome sequencing of extended-spectrum β-lactamase genes in Enterobacteriaceae isolates from Nigeria
Source: PLoS One. 2020 Apr 14;15(4):e0231146. doi: 10.1371/journal.pone.0231146 (PMC7156064; doi:10.1371/journal.pone.0231146)
Supplement: Attachment — Submitted filename: rebuttal letter 1.docx [file pone.0231146.s006.docx]

Reviewer No1

Line 36. Enterobacteriaceae

**Correction has been effected on the revised manuscript**

Line 38. Specie of Providencia spp

**The revised manuscript has been updated. We decided to strike out *Providencia spp* as part of the reported ESBL isolates as it was sensitive to the ESBL phenotypic screening but we detected CTX-M-15 by sequencing. However during our submission of the sequences to NCBI also the sequence had issues that were challenging to resolve so we decided not to include it in the study anymore. The genome submission to NCBI gave a better insight with regards to the isolates.**

Line 88. Repeated “Fig 1. Figure showing sample size of Enterobacteriaceae isolates provided by three Nigerian hospitals”

**Correction has been effected on the revised manuscript.**

Line 90. Incomplete sentence

**Sentence has been updated in the revised manuscript.**

Line 94. patients

**Correction has been effected on the revised manuscript**

According to the answer The ESBL screening and confirmation performed well in strains with ESBL and carbapenemase. Please consider that strains with ampC and carbapenemases can affect the performance of ESBL screening test

**Noted. The 60 ESBL isolates presented including the ones with carbapenemases were positive to both the phenotypic screening for ESBL and the sequencing. Alongside other resistance genes only carbapenemases were detected in the ESBL isolates. No ampC was detected in any of the ESBL isolates.**

Line 87. Please include that the 3 hospitals are tertiary healthcare institutions, number of beds

**Revised manuscript has been updated**

Line 116- 128 Consider to rewrite the paragraph. There are words repeated “manufacturer’s instructions”

**Paragraph has been revised.**

Line 195. Specie of Providencia spp

**Manuscript has been revised. *Providencia* isolate is no longer included in the study**. **Reasons have been stated earlier in the rebuttal letter.**

Line 233. The study concludes a probable clonal spread of the ESBL strains especially intra-hospital. Does WGS gave you some evidence? If not please consider the limitations.

**Using the data obtained from the MLST assignment and plasmid incompatibility grouping, the distribution of the sequence types and the plasmid replicon types especially the predominant ones of the ESBL isolates across the three hospitals suggests a probable clonal spread of the ESBL strains both intra and inter-hospital. A limitation of this study however is to conclude on clonal bacterial transmission between ESBL *Enterobacteriaceae* isolates that included seven groups of different organisms.**

The findings in the study can support a better conclusion in the abstract and the manuscript

**Conclusion in the abstract and manuscript have been revised**

**Reviewer No 2**

**Line 77.** Please use additional bibliography to support this statement

**Additional bibliography has been added to the revised manuscript.**

Line 79. Please check the bibliography number 21

**Bibliography no 21 has been revised**

Line 87. Include that are tertiary hospitals. As a comment, Figure 1 does not improve the manuscript

**The manuscript has been revised indicating the hospitals are tertiary healthcare institutions. The figure was included in response to another reviewer’s comment from the previous revision.**

All isolates were obtained from hospitalized patients? Please specify if not
Line 134: specify which isolates were further studied with MLST. The results are just for K.pneumoniae, E.coli and E. cloacae?

**Isolates were obtained from samples of both in-patients and out-patients of the three different hospitals. All ESBL isolates were further studied with MLST. Sequence type numbers were not available for other isolates not presented. Some isolates had unknown sequence types which are submitted for curation of sequence type number.**

Results
Piperacillin-tazobactam for K. pneumonia n = 9

**Correction has been updated in the revised manuscript**

results about imcompatibility groups and ST shoould be presented in a resume table. The current version is hard to read

**Table has been revised. Result about incompatibility groups and ST has been represented in a separate table.**

Discussion
The manuscript does not highlight well their results. Include some data about ESBL in other countries from Africa and compare them.

**Discussion has been revised highlighting the results properly. Data about ESBL in other countries from Africa have been included and compared with the study**
